# Pediatric Acute Respiratory Distress Syndrome: Fibrosis versus Repair

**DOI:** 10.3389/fped.2016.00028

**Published:** 2016-03-30

**Authors:** Daniel Im, Wei Shi, Barbara Driscoll

**Affiliations:** ^1^Pediatric Critical Care Medicine, Department of Pediatrics, The Saban Research Institute, Children’s Hospital Los Angeles, University of Southern California, Los Angeles, CA, USA; ^2^Developmental Biology and Regenerative Medicine Program, Department of Surgery, The Saban Research Institute, Children’s Hospital Los Angeles, University of Southern California, Los Angeles, CA, USA

**Keywords:** acute lung injury, pediatric pulmonary fibrosis, lung injury repair, pediatric lung inflammation, pediatric respiratory distress syndrome, surfactant therapy

## Abstract

Clinical and basic experimental approaches to pediatric acute lung injury (ALI), including acute respiratory distress syndrome (ARDS), have historically focused on acute care and management of the patient. Additional efforts have focused on the etiology of pediatric ALI and ARDS, clinically defined as diffuse, bilateral diseases of the lung that compromise function leading to severe hypoxemia within 7 days of defined insult. Insults can include ancillary events related to prematurity, can follow trauma and/or transfusion, or can present as sequelae of pulmonary infections and cardiovascular disease and/or injury. Pediatric ALI/ARDS remains one of the leading causes of infant and childhood morbidity and mortality, particularly in the developing world. Though incidence is relatively low, ranging from 2.9 to 9.5 cases/100,000 patients/year, mortality remains high, approaching 35% in some studies. However, this is a significant decrease from the historical mortality rate of over 50%. Several decades of advances in acute management and treatment, as well as better understanding of approaches to ventilation, oxygenation, and surfactant regulation have contributed to improvements in patient recovery. As such, there is a burgeoning interest in the long-term impact of pediatric ALI/ARDS. Chronic pulmonary deficiencies in survivors appear to be caused by inappropriate injury repair, with fibrosis and predisposition to emphysema arising as irreversible secondary events that can severely compromise pulmonary development and function, as well as the overall health of the patient. In this chapter, the long-term effectiveness of current treatments will be examined, as will the potential efficacy of novel, acute, and long-term therapies that support repair and delay or even impede the onset of secondary events, including fibrosis.

## Introduction

Since first described by Ashbaugh and colleagues in 1967 as “Adult Respiratory Distress Syndrome in Children,” Pediatric Acute Respiratory Distress Syndrome (Pediatric ARDS) has been recognized as a distinct syndrome, with hallmarks and outcomes that differ from those of patients suffering from adult ARDS or neonatal RDS (1–3). Pediatric ARDS is a syndrome of non-cardiogenic pulmonary edema with clinical hallmarks including hypoxemia, bilateral radiographic opacities, increased venous admixture, decreased functional residual capacity, increased physiological deadspace, and decreased lung compliance. Early studies singled out lung surfactant deficiency that can be caused by a large variety of pulmonary and extra-pulmonary insults as an underlying factor in Pediatric ARDS, but the pathophysiology has since been recognized as much more complex (3). Key precipitating events are (1) activation of an unruly inflammatory milieu, which leads to (2) injury to the alveolar epithelial-endothelial permeability barrier, followed by (3) impaired alveolar fluid clearance. This fundamental change in pulmonary homeostasis can be caused by injury to either the alveolar or the capillary epithelium (4). The time course of lung structural damage in Pediatric ARDS has been classically thought to occur in three phases: an inflammatory exudative phase, characterized by diffuse alveolar injury with necrosis of alveolar type I cells, increase in vascular permeability, and an influx of inflammatory cells; a proliferative phase, starting approximately 72 h after the initial insult and lasting approximately 7 days, associated with alveolar epithelial type II (AEC2) repair; mesenchymal cell (interstitial fibroblast and myofibroblast) migration, proliferation, and secretion of extracellular matrix proteins, such as collagen; and a fibrotic phase, characterized by significant upregulation of collagen synthesis and chronic remodeling (4–10). Conventional treatments include ventilatory support, fluid clearance, and/or, in the most extreme cases, extra-corporeal membrane oxygenation (ECMO). Exogenous surfactant administration, prone positioning, and anti-inflammatory approaches, including administration of corticosteroids, may also be used, though consensus on the efficacy of these therapies is not universal, especially in children, for whom data are sparse (3, 11–14). Pediatric survivors with a history of ARDS exhibit pulmonary function characteristic of restrictive lung disease, though the severity of this functional deficit may fade over time, indicating a capacity for repair in a significant subset of patients. It has come to be understood that the timeline of disease progression in Pediatric ARDS is not as clearly as demarcated as originally described and outcomes can vary, dependent on both individual response and course of treatment. In this chapter, the complex cellular and molecular events that determine whether outcome features a more reparative or a more fibrotic profile will be examined. A better understanding of how fibrosis is precipitated and how repair might be facilitated in pediatric patients is critical for addressing the pulmonary fibrotic outcome of Pediatric ARDS, which is associated worldwide with significant levels of morbidity and mortality.

## Pathophysiology and Outcomes: Pediatric ARDS Versus Adult ARDS and Neonatal RDS

Classically, Neonatal RDS has been described as a disease of lung immaturity with consequent surfactant insufficiency, which responds well to supplementary surfactant treatment. This approach has substantially changed outcomes for premature newborns and is one of the great success stories of modern neonatal medicine. In contrast, treatment of adult ARDS with supplementary surfactant has not been as successful (15–18). Studies by Willson and colleagues on pediatric patients show that children, while not as responsive as neonates, are still much more responsive to surfactant replacement therapy than adults. In a number of studies, Pediatric ARDS patients show improvement following administration of synthetic exogenous surfactants, particularly when the formula is stabilized (19–22). These differences in response are presumably due to the etiology of the syndrome in neonatal versus pediatric versus adult patients. In neonata patients suffering from prematurity, surfactant therapy not only supports pulmonary function but may also aid in lung maturation. In pediatric and adult lung, in which lungs have matured or are in the latter stages of maturing and which have no prior surfactant compromise, exogenous surfactant may acutely support lung function, but may not have as striking an impact on already mature epithelium. Although ARDS patients do suffer from diminished surfactant levels due to trauma to the surfactant secreting AEC2 population and/or sequestration of surfactant by substances present following edema or inflammation, the pathophysiology of the syndrome is clearly more complex than can be ascribed to this loss. Pediatric ARDS can occur in children with no pre-existing illnesses, but occurrence and mortality are both significantly higher in those with serious underlying conditions, including oncologic diagnoses and especially immune deficiency (23). The syndrome in children also differs from adult disease in outcome, again pointing to potential, underlying differences in pathophysiology. Overall mortality in Pediatric ARDS patients is much lower than that for adult patients. In fact, a subset of pediatric patients show a remarkable capacity for repair, whereas in Adult ARDS survivors, lung function is more often reduced long-term, with permanent alveolar simplification and fibrosis presumably due to regenerative failure. While pediatric survivors can be compromised by exertional dyspnea, hypoxemia, and, in some cases, persistent peripheral airway disease, these patients as a whole appear to possess a notable capacity to recover alveolar units destroyed by trauma (24–26).

Pediatric ARDS develops following a broad range of injuries, when breakdown of the alveolar permeability barrier leads to an influx of proteins and fluids across lung epithelium and endothelium and accumulation of protein-rich inflammatory fluid in the normally fluid-free alveolar space. Altered alveolar permeability and fluid accumulation occur in the setting of inflammation, featuring inappropriate activation of leukocytes and platelets, and uncontrolled activation of coagulation pathways. Solutes and large molecules, such as albumin, enter the alveolar space, creating a highly proteinaceous edema. Significant concentrations of cytokines [e.g., interleukin (IL)-1, IL-8, and tumor necrosis factor (TNF)-α] and lipid mediators (e.g., leukotriene B4) are also present. In response to pro-inflammatory mediators, leukocytes – especially neutrophils – migrate into the pulmonary interstitium and alveoli (4). The presence of protein, fibrinogen, and fibrin degradation products in the edema fluid due to impairment of alveolar fluid clearance leads to surfactant degradation and a subsequent increase in alveolar surface tension. A resulting fall in pulmonary compliance and increased alveolar instability ultimately lead to areas of atelectasis and significant pulmonary dysfunction.

Following acute lung injury (ALI), not only inflammatory cytokines but also growth-promoting agents, especially in pediatric patients that are still developing (e.g., PDGF, TGF-α, TGF-β), are released systemically and locally, as well as activated in the disrupted alveolar structure, which initiate repair processes in response to the injury. It appears that in a subset of pediatric patients, this response can occur swiftly in response to trauma. One retrospective study showed that Pediatric ARDS survivors that exhibit immediate, positive changes in response to treatment over the first 3 days following onset have much faster, more complete reparative outcomes compared to those who do not respond as quickly (27). How a slower response to treatment might contribute to fibrosis in survivors is still under investigation. Inappropriate mesenchymal cell proliferation and extracellular matrix production (known downstream responses to TGF-α and TGF-β, in particular) due to severe or repeated epithelial cell damage have been shown in both animal models and in human patients to eventually evolve into alveolar fibrosis (28, 29). As intra-alveolar fibrosis evolves, infiltrating leukocytes decrease and are replaced by increased numbers of mononuclear phagocytes within the alveolar wall and airspace. Additionally, a variety of signals that are capable of stimulating alveolar macrophage growth factor release (e.g., endotoxin, activated complement components) are present in the distal lung under most of the circumstances associated with acute pediatric lung injury. It may be that slower patient responses allow a cascade of signaling events to occur that promotes long-term, pro-fibrotic remodeling. Though this hypothesis has yet to be completely tested, multiple studies in animal models and on cadaveric human tissue have demonstrated potential mechanisms and intervention points for the development of fibrosis following ALI resulting in Pediatric ARDS.

This fibroproliferative response, which precludes alveolar repair, is regulated by many factors (30, 31). Madtes et al. described a role for the epithelial and mesenchymal cell mitogen transforming growth factor (TGF)-α in the fibroproliferative response in ARDS and have shown that significant levels of TGF-α and procollagen peptide (PCP) III, a biological marker of fibroproliferation, are present in the BAL fluid of ARDS patients. Armstrong et al. noted that BAL fluid from patients with ARDS showed an early shift in the balance between type I collagen synthesis and degradation by collagenase, which favors development of fibrosis (32). As early as 36 h following the onset of injury, this fibrotic phase begins with extensive remodeling of sparsely cellular and collagenous tissue within the distal lung (30). Early morphologic examination of the lungs of patients that die from ALI reveals a common anatomic theme, in which a marked accumulation of mesenchymal cells and their connective tissue products appear in the alveolar airspace and in the walls of intra-acinal microvessels, i.e., an acute fibroproliferative response (33). On gross examination, the lungs of patients with ARDS appear heavy and edematous. The surface appears violaceous and hemorrhagic fluid exudes from the cut pleural surface (4). Microscopically, within hours of the onset of lung injury, the air-lung interface is denuded as type 1 epithelial cells (AEC1) die; within a few days after the inciting injury, there are intra-alveolar accumulations of mesenchymal cells, macrophages, and inflammatory cells (33–35). The interstitium becomes expanded with an increasing number of mesenchymal cells and numerous collagen fibrils and elastic fibers (35). The epithelial basement membrane becomes compromised with gaps of denuded areas, leaving the disrupted interstitium in direct communication with the alveolar airspace. Hyaline membranes form in the absence of alveolar epithelium. Vascular obliteration by microthrombi and fibrocellular proliferation also occurs. Activated myofibroblasts from the interstitium migrate into the alveolar airspace through the gaps in the compromised basement membrane and attach to the luminal surface of the damaged basement membrane (36). While presumably an attempt at repair, blockage of further damage, and/or a valiant effort to prevent drowning due to vascular leak, once myofibroblasts proliferate and persist, the excessive production of abnormal extracellular matrices ultimately obliterates the gas exchange unit. Immunohistochemical studies have shown increased amounts of fibronectin in the airspaces of ARDS patients. Biochemical analysis has also shown an accumulation of collagens type I and III in the expanded interstitium, with type III collagen predominantly present in the early stages following lung injury and type I collagen present at a later stage (36, 37).

Thus, the fibrotic phase of Pediatric ARDS is identified by the deposition of excess collagen and other extracellular matrix material and is associated with intra-alveolar, alveolar septal wall and alveolar ductal fibrosis as there is an attempt by the body to recreate the alveolar basement membrane that overlies collapsed and atelectatic regions of the lung (30, 38). Together, these pathologic changes result in the impaired lung physiology characteristic of Pediatric ARDS, which includes decreased functional residual capacity, diminished compliance accompanied by an increase in the work of breathing, increased deadspace and shunt fraction, and impaired gas exchange (39–41). The persistence of fibrosis in adult ARDS survivors has been documented to 2 years or more, by biopsy (25). Data on pediatric ARDS survivors show much more potential for repair, though this has mainly been demonstrated indirectly, by follow up that includes spirometry. Conversely, pediatric patients that respond poorly to initial treatments, even if they do not succumb, exhibit a problematic course of recovery that may require prolonged ventilation that can last much longer than that administered to quickly responding pediatric patients and adult survivors (23, 42). Unfortunately, this mechanical intervention has a well-known capacity to feed forward to create further injury via mechanical stretch and mesenchymal activation, thereby exacerbating the development of fibrosis.

## Mechanical Ventilator Associated Fibrosis

It is important to consider the role of mechanical ventilation in the development of fibrosis in Pediatric ARDS patients. Protective mechanical ventilation is considered the most important supportive therapy for pediatric patients with ARDS. This approach was first validated by a multicenter randomized trial on Adult ARDS patients conducted by the ARDS Network under the auspices of the National Institutes of Health (43). Subsequent trials for Pediatric ARDS patients have generated some equivocal data, but the general agreement in the field is that ventilator support, including high-frequency oscillating ventilation (HFOV), especially if applied early in the disease course, has a beneficial impact (11, 12, 42). However, mechanical ventilation can also induce or aggravate lung injury, referred to as ventilator-induced lung injury (VILI) (5), to which the smaller, still maturing lungs of children can be particularly susceptible. The level of antioxidants, a critical component of lung homeostasis, can be compromised in Pediatric ARDS by alterations in both cell populations and the balance of proteins and water in interstitial spaces, leading to increased susceptibility to oxygen toxicity induced by forced ventilation. Pro-inflammatory and profibrotic responses may become persistent or uncontrolled during mechanical ventilation. Notably, recent ventilation strategies aimed at decreasing lung stretch has significantly decreased mortality (11, 12). However, the morbidity of mechanical ventilation remains as ventilation at high transpulmonary pressures leads to “barotrauma” and alveolar overdistention leads to “volutrauma,” both characterized by increased alveolar-capillary leak and pulmonary edema (44, 45). Repeated opening and collapsing of delicate alveolar spaces at abnormally high pressures contributes to injury via a mechanism called “atelectrauma” (46). In all, there is an emerging evidence demonstrating that barotrauma, volutrauma, and/or atelectrauma from mechanical ventilation influence the course of lung remodeling in experimental models, which provide insight into the pathologies observed in over-ventilated Pediatric ARDS patients (47, 48).

In isolated rat lung and in lung parenchymal strips, mechanical stretch results in enhanced PCPIII expression. Application of recruitment maneuvers associated with atelectasis has also been shown to increase PCPIII expression in animal models of ARDS. In open-chest rabbits, mechanical ventilation with a high-positive end expiratory pressure led to greater gene expression of PCPIII, procollagen IV, fibronectin, and TGF-β1, the classical growth factor for fibrosis formation (49–51). In contrast, ventilation with a low PEEP did not stimulate expression of these genes. Furthermore, mechanical ventilation causes injury not only by structural disruption of the lung but also by induction of inflammatory responses associated with mediator release, which can worsen lung injury and potentially cause systemic organ dysfunction (52–55). The physical forces generated during mechanical ventilation can induce the release and/or activation of cytokines, chemokines, and growth factors in lungs, which together with inflammatory cell recruitment, may play a significant role in the progression from injury to fibroproliferation in pediatric patients. This altered condition in the lung presumably impacts any ability to repair. It can also alter the homeostatic profile and function of the lung even after repair, making once-damaged lungs especially vulnerable to further injury. Taken together, these studies suggest that atelectasis and alveolar overdistention due to mechanical ventilation are harmful and can lead to or exacerbate the development of fibrosis, while careful ventilation management in responsive patients, can support repair.

## Repair

Pediatric lungs in general have a substantial potential for repair and recovery after ARDS. In major treatment centers, a majority of pediatric patients recover from ARDS. In community treatment settings, as well as globally, this percentage is lower, however, indicating that a critical, early time frame exists for effective intervention. This hypothesis is reinforced by the observation that pediatric patients that respond rapidly to treatment and/or recover by 6 months following the initial insult often recover completely (23). However, pediatric lung injury can have divergent outcomes, with the appearance of fibrosing alveolitis in some patients versus others with seemingly equivalent degrees of lung injury that resolve the inflammation without evidence of fibrosis. The underlying molecular basis of these different outcomes is unclear. Tissue repair involves a variety of mechanisms including edema reabsorption, resolution of inflammation, and cell proliferation in order to repopulate the alveolar epithelium. Strategies that support these natural mechanisms of repair are currently under study using both animal models and clinical trials (Table 1). Unfortunately, few of these present data on long-term outcomes for Pediatric ARDS patients. Thus, a better understanding of the mechanisms underlying repair in the juvenile lung, and how they may be supported by conventional and experimental therapies, is highly warranted.

**Table 1:** **Pediatric ARDS Therapies (NB: impact on repair versus fibrosis as a long-term outcome is currently unknown, even for animal studies, for all treatments)**.

Therapy	Data from animal models (adult or neonatal)	Data from *pediatric* pilot and experimental studies and clinical trials	In use routinely or experimentally in *pediatric* patients
Ventilatory support	(56–58)	(3, 11, 12, 43, 59–61)	Yes^b^
Fluid clearance	(62)	(59, 63–65)	Yes^a,b^
ECMO	(66)	(67, 68)	Yes^b^
Surfactant replacement therapy using synthetic and animal-derived surfactants, ±inhaled nitric oxide	(57, 69–77), reviewed in Ref. (16)	(19–21, 78–92) reviewed in Ref. (16, 22)	Yes^a,c,d^
Corticosteroids and other anti-inflammatories and fluid clearance agents	(93–95)	(96)^e,f^	Yes^a,c^
Gene therapy for fluid clearance and anti-inflammatory impact	(97–105), reviewed in Ref. (106)	ND^e^	No
Mesenchymal (or other) stem cells	Reviewed in Ref. (107)	ND^e^	No
Growth factors (epidermal and fibroblast growth factor family members)	(108–113)	ND	No

*^a^Used in conjunction with ventilatory support*.

*^b^Recommended by the Pediatric Acute Lung Injury Consensus Conference Group*.

*^c^Not recommended by the Pediatric Acute Lung Injury Consensus Conference Group but may be considered in severe cases*.

*^d^Clinical trials have curtailed use of certain synthetic surfactants that have shown lesser efficacy than animal-derived surfactants*.

*^e^Trials, safety, pilot, or experimental studies performed in adult patients; few or no studies in pediatric patients*.

*^f^Trials, safety, pilot, or experimental studies performed in neonatal or premature patients; few or no studies in pediatric patients*.

The inflammatory response in ARDS is a complex sequence of events that requires the interplay between several immune mediators (114, 115). In the most reparative scenario, anti-inflammatory cytokines (e.g., IL-10) are released as a negative feedback mechanism. When pro-inflammatory pathways are downregulated, these anti-inflammatory mediators can then decrease cytokine expression. Although anti-inflammatory mediators are involved in limiting inflammation, there are also pro-resolution mediators that act to mitigate inflammation and restore tissue homeostasis without causing immune suppression. Pro-resolution mediators include several classes of signaling molecules generated from polyunsaturated fatty acids: lipoxins, resolvins, and protectins (4). These agents appear to signal the recruitment of macrophages, the phagocytosis of apoptoic neutrophils, and the secretion of molecules that can act as anti-inflammatory agents at later stages of recovery, such as IL-10 and TGF-β. Apoptosis of inflammatory cells, mainly neutrophils, has also been shown when pro-survival signals such as granulocyte-colony stimulating factor (G-CSF), disappear. Alveolar macrophages have also been shown to play a role in this phase by engulfing apoptotic cells (114). Therefore, modulation of the inflammatory milieu using corticosteroids, direct administration of anti-inflammatory cytokines, or administration of recombinant viruses or modified stem cells that secrete immunomodulatory molecules have all been proposed to address this aspect of Pediatric ARDS. As yet, with the exception of steroid administration, these approaches have yet to move beyond the experimental phase.

The regeneration of the alveolar structure requires the proliferation and differentiation of AEC2 progenitors into AEC1 pneumocytes (116). It was recently established that the oxygen environment has a significant impact on AEC2 stem cell capacity during development, which may also occur following injury (117). This may particularly hold true in the case of Pediatric ARDS, where oxygenation is disrupted by trauma and artificially modulated by ventilation. Repair of the epithelium is a complex process that involves epithelial cell spreading and migration, as well as proliferation and differentiation of the stem cell population. It is this critical response that may underlie the difference between Pediatric and Adult ARDS patients in their capacity for both initial recovery and long-term return to normal lung function. Given the more robust nature of chronologically younger stem cells, therapeutic approaches that support stem cell function should be a priority for pediatric patients as newer treatments are developed. During the early stages of epithelial repair, epithelial progenitor cells migrate along the underlying matrix that is, in turn, remodeled during the repair process. Growth factors (EGF, KGF, and HGF) that act through tyrosine-kinase receptors, promote cell proliferation (116), and exogenous administration of KGF *in vivo* has been shown to speed this process [(118–120), reviewed in Ref. (121)]. Multiple other molecules that support lung progenitor cells have been extensively studied, *ex vivo*, in isolated primary human and animal cells in culture and in non-transformed human lung cell lines. These include chemokine receptors, anti-inflammatory ILs, eicosinoids, modulators of integrins, and matrix metalloproteinases (MMPs) to aid spreading and wound closure while blocking excessive collagen deposition, modulation of Rho GTPases and MAP kinases, modulation of STAT, PTEN, and PI3 kinase signaling, all critical for a broad range of stem cell functions, modulators of epithelial-mesenchymal transition, including Wnt1, TGF-ß, and E-cadherin (121). As yet, few of these attempts to modify and/or support epithelial stem cell-directed repair have been translated to even comprehensive *in vivo* studies, and clinical trials have yet to be achieved. Likewise, use of gene therapy or modified stem cell therapy approaches that modulate the function of these molecules and pathways within the context of Pediatric ARDS are still highly speculative approaches, with limited data available from laboratory studies and contraindications from experimental adult human trials (106). Additional studies have attempted to determine the regenerative capacity of subsets of endogenous progenitor cells in the absence of exogenous support, though these studies too are at a very early stage and recapitulation using human lung stem cell and progenitor populations has been slow. Endogenous progenitor cells include both resident epithelial and mesenchymal stem cells and bone marrow-derived mesenchymal cells. Recent studies in rodent models suggest that there may be other lung progenitor cells involved in the repair of the lung epithelium, including club cells, integrin α6β4 alveolar epithelial cells, and Scgb1a1-expressing cells and AEC1 themselves (122–124). However, the majority of data to date on lung epithelial response to injury has been generated by focusing on the role of AEC2. AEC2 classically represent the resident stem cells that can proliferate after injury and transdifferentiate into AEC1. In experimental models, AEC2 have been shown to migrate, proliferate, and differentiate into AEC1 (125). A recently developed experimental mouse model of extreme (>80%) AEC2 depletion that results, at both acute and long-term follow up stages, in a distal lung that phenotypically resembles that of ARDS patients that suffer fibrotic outcomes, indicating the essential role for these cells in alveolar homeostasis (126). Furthermore, newer data show that if this depletion is induced in juvenile mice, repair is more robust and outcome is much more favorable. These data indicate that resident distal lung stem cell populations harbor highly functional stem cells capable of efficient repair under favorable circumstances.

In addition to its stem cell capacity, the AEC2 population also plays a critical role in surfactant production. Though surfactant therapy is often considered an acute response to the problem of surfactant deficiency that occurs within the early stages of ARDS, there are experimental data to show that the presence of surfactant itself is critical for maintaining AEC2 homeostasis (127–129). Additional data from animal models and studies using isolated patient cells indicate that a robust, functional AEC2 population and the presence of normal surfactant levels function as a defense against development of fibrosis (130–133). Thus, surfactant therapy for Pediatric ARDS, while it has still not achieved the level of success when used for Neonatal RDS, is still considered a promising and important mechanism for supporting repair (16, 22), particularly when used in conjunction with standard ventilation approaches. This is especially true for ARDS caused by direct pulmonary insults (infection, trauma, radiation, drowning, toxin exposure), where the pulmonary epithelium, including AEC2 is the main target, versus indirect causes (sepsis, burns, shock, transfusions), where damage to the alveolar capillary epithelium may play a greater role. Thus, this latter variety of Pediatric ARDS may not be as responsive to alveolar epithelial supportive therapy (21, 78).

Interestingly, there is also evidence that populations of pulmonary and even extra-pulmonary-derived stem cells that are present in the circulation in response to ARDS may play a role in alveolar regeneration. Lung mesenchymal cells are activated after lung injury and, in addition to collagen synthesis, may secrete growth factors and modulate the immune system by secreting anti-inflammatory cytokines (134). Clinical observations indicate the presence of endothelial progenitor cells post-ALI that may contribute to repair, though findings so far have been limited to adult and neonatal patients and to animal models (107). In contrast, a study by Bui and colleagues pediatric patients being treated by ECMO, including some suffering from Pediatric ARDS, showed that distinctive populations of stem-like cells of hematopoietic, mesenchymal, and epithelial lineages could be isolated from ECMO circuits, indicating that novel stem-like cell populations may be mobilized into the circulation by severe lung injury (135). Interestingly, the lineage prevalence and stem-like characteristics of these cells differed with patient age, again supporting the observation that pediatric ARDS patients respond in ways unique to their chronological age, in ways that differ from Neonatal RDS and Adult ARDS patients. An additional finding from this study was that a significant number of stem-like cells, especially those exhibiting characteristics of epithelial progenitors, were isolated from ECMO circuits very early in course of treatment, which correlates with the observation that those patients that respond quickly to treatment exhibit better outcomes. Taken together, these data indicate that early interventions that support endogenous stem cell function and take advantage of the patients’ own response to pulmonary insult should be a focus of future treatments. In addition, conventional treatments that support reparative outcomes should also be more thoroughly investigated and augmented where possible.

One conventional treatment that impacts surfactant function, as well as the general integrity of the alveolar space, is fluid reduction. In order to restore normal respiratory function post-injury, collagen scars formed to preserve alveolar integrity and prevent drowning due to edema caused by vascular leak. These scars must be processed and removed by MMPs, a family of enzymes that digest extracellular fibers (136). One of the most important sources of MMPs is inflammatory cells, such as neutrophils and macrophages. Thus, ironically, the inflammatory response that can cause acute injury is important during resolution stages for adequate lung repair. MMPs represent one of the links between these two phenomena of inflammation and repair. MMPs are upregulated during the repair process and appear to be involved in facilitating cellular migration and the remodeling of the ECM (121). Studies in animal and tissue culture models indicate that migration and proliferation of epithelial progenitor cells are regulated by soluble factors released in response to lung injury. These factors include members of the epidermal growth factor family (EGF and TGF-α) and fibroblast growth factor family (HGF, KGF, FGF-10). Once the alveolar permeability barrier is re-established, removal of lung edema occurs via movement of water out of the airspaces through aquaporins (water channels) in AEC2, which is driven by the active transport of sodium and chloride through specific epithelial cell ion channels (eNAC and CFTR) (101, 106, 137–140). This endogenous repair function can be augmented by mechanical suction, with or without saline lavage (140, 141). More recent studies have focused on pharmacological stimulation of epithelial ion channels, though these studies, again, are still at the experimental stage.

## Summary

Since identification of the pathways involved in lung injury, most of the literature has been focused on the use of therapies aimed at truncating the inflammatory response. However, recent studies have, and future studies should, focus on enhancing the repair process. Using approaches that address both acute inflammation and support repair in combination has also been given recent consideration, as each may address particular pathways that contribute to shifting long-term outcomes from pro-fibrotic to pro-repair (16, 142, 143). While Pediatric ARDS survivors exhibit an obstructive disease phenotype, at least during the acute phase and within the first year following recovery, significant long-term improvement in some patients has been documented. It is therefore imperative to consider bold strategies that could support repair during this period. A variety of approaches have been suggested, ranging from the use of biocompatible materials or cells to bolster remodeling to the therapeutic use of mediators aimed at promoting normal cell proliferation, migration, and differentiation. Because the mechanisms that cause tissue disruption and inflammation in the early phase of lung injury also contribute to repair and matrix remodeling later on, a fundamental key to future therapy is impeding the onset of fibrosis. Thus, therapies that disrupt pro-inflammatory pathways, such as MMP inhibition, may have a prophylactic value. Growth factors, exogenous stem cells, or drugs that promote matrix remodeling could also improve both the short- and long-term prognosis of patients with ARDS. Knowledge of the mediators involved in tissue repair and re-establishment of normal development and lung homeostasis could lead to new therapeutic strategies being applied of pediatric patients after the initial insult has been controlled. In fact, given the much higher potential for regeneration and repair in children, future experimental and clinical studies should be directed toward therapies for these patients that support effective repair, proper regeneration, and a return to homeostasis.

## Author Contributions

DI drafted the paper and added additional research. WS contributed to the writing of the paper and added additional research. BD wrote the abstract, contributed to the writing of the paper, and contributed initial and additional research.

## Conflict of Interest Statement

The authors declare that the research was conducted in the absence of any commercial or financial relationships that could be construed as a potential conflict of interest.

## References

[1] Ashbaugh DG Bielow DB Petty TL Levine BE Acute respiratory distress in adults. Lancet (1967) 2:319–23. 10.1016/S0140-6736(67)90168-7 4143721

[2] Paulson TE Spear RM Peterson BM. New concepts in the treatment of children with acute respiratory distress syndrome. J Pediatr (1995) 127:163–75. 10.1016/S0022-3476(95)70291-1 7636639

[3] Cornfield DN. Acute respiratory distress syndrome in children: physiology and management. Curr Opin Pediatr (2013) 25:338–43. 10.1097/MOP.0b013e328360bbe7 23657244

[4] Sapru A Flori H Quasney MW Dahmer MK Pediatric Acute Lung Injury Consensus Conference Group Pathobiology of acute respiratory distress syndrome. Pediatr Crit Care Med (2015) 16:S6–22. 10.1097/PCC.0000000000000431 26035365

[5] Cabrera-Benitez NE Laffey JG Parotto M Spieth PM Villar J Zhang H Mechanical ventilation-associated lung fibrosis in acute respiraotry distress syndrome. Anesthesiology (2014) 121:189–98. 10.1097/ALN.0000000000000264 24732023 PMC4991945

[6] Pastor CM Matthay MA Frossard JL Pancreatitis-associated acute lung injury: new insights. Chest (2003) 124:2341–51. 10.1378/chest.124.6.2341 14665518

[7] Rocco PR Negri EM Kurtz PM Vasconcellos FP Silva GH Capelozzi VL Lung tissue mechanics and extracellular matrix remodeling in acute lung injury. Am J Respir Crit Care Med (2001) 164:1067–71. 10.1164/ajrccm.164.6.2007062 11587998

[8] Zapol WM Trelstad RL Coffey JW Tsai I Salvador RA Pulmonary fibrosis in severe acute respiratory failure. Am Rev Respir Dis (1979) 119:547–54. 443627 10.1164/arrd.1979.119.4.547

[9] Raghu G Striker LJ Hudson LD Striker GE Extracellular matrix in normal and fibrotic human lungs. Am Rev Respir Dis (1985) 131:281–9. 3882034 10.1164/arrd.1985.131.2.281

[10] Curley GF Contreras M Higgins B O’Kane C McAuley DF O’Toole D Evolution of the inflammatory and fibroproliferative responses during resolution and repair after ventilator-induced lung injury in the rat. Anesthesiology (2011) 115:1022–32. 10.1097/ALN.0b013e31823422c9 21952251

[11] Pediatric Acute Lung Injury Consensus Conference Group. Pediatric acute respiratory distress syndrome: consensus recommendations from the pediatric acute lung injury consensus conference. Pediatr Crit Care Med (2015) 5:428–39. 10.1097/PCC.0000000000000350 PMC5253180 25647235

[12] Tamburro RF Kneyber MCJ Pediatric Acute Lung Injury Consensus Conference Group. Pulmonary specific ancillary treatment for pediatric acute respiratory distress syndrome: proceedings from the pediatric acute lung injury consensus conference. Pediatr Crit Care Med (2015) 16:S61–72. 10.1097/PCC.0000000000000434 26035366

[13] Schneider J Sweberg T. Acute respiratory failure. Crit Care Clin (2013) 29:167–83. 10.1016/j.ccc.2012.12.004 23537670

[14] Mok YH Lee JH Rehder KJ Turner DA Adjunctive treatments in pediatric acute respiratory distress syndrome. Expert Rev Respir Med (2014) 8:703–16. 10.1586/17476348.2014.948854 25119574

[15] Taeusch HW. Treatment of acute (adult) respiratory distress syndrome: the Holy Grail of surfactant therapy. Biol Neonate (2000) 77:2–8. 10.1159/000047050 10828579

[16] Raghavendran K Willson D Notter RH Surfactant therapy of ALI and ARDS. Crit Care Clin (2011) 27:525–59. 10.1016/j.ccc.2011.04.005 21742216 PMC3153076

[17] Anzueto A Baughman RP Guntupalli KK Weg JG Wiedemann HP Raventós AA Aerosolized surfactant in adults with sepsis-induced acute respiratory distress syndrome. N Engl J Med (1996) 334:1417–21. 10.1056/NEJM199605303342201 8618579

[18] Gregory TJ Steinberg KP Spragg R Gadek JE Hyers TM Longmore WJ Bovine surfactant therapy for patients with acute respiratory distress syndrome. Am J Respir Crit Care Med (1997) 155:109–31. 10.1164/ajrccm.155.4.9105072 9105072

[19] Willson DF Jiao JH Bauman LA Zaritsky A Craft H Dockery K Calf lung surfactant extract in acute hypoxemic respiratory failure in children. Crit Care Med (1996) 24:1316–22. 10.1097/00003246-199608000-00008 8706485

[20] Willson DF Bauman LA Zaritsky A Dockery K James RL Stat M Instillation of calf lung surfactant extract (calfactant) is beneficial in pediatric acute hypoxemic respiratory failure. Crit Care Med (1999) 27:188–95. 10.1097/00003246-199901000-00050 9934915

[21] Willson DF Thomas NJ Markovitz BP Bauman LA DiCarlo JV Pon S Effect of exogenous surfactant (calfactant) in pediatric acute lung injury. JAMA (2005) 293:470–6. 10.1001/jama.293.4.470 15671432

[22] Willson DF Chess PR Notter RH. Surfactant for pediatric acute lung injury. Pediatr Clin North Am (2008) 55:545–62. 10.1016/j.pcl.2008.02.016 18501754 PMC4275446

[23] Redding GJ Current concepts in adult respiratory distress syndrome in children. Curr Opin Pediatr (2001) 13:261–6. 10.1097/00008480-200106000-00009 11389362

[24] Fanconi S Kraemer R Weber J Tschaeppeler H Pfenninger J. Long-term sequelae in children surviving adult respiratory distress syndrome. J Pediatr (1985) 106:218–22. 10.1016/S0022-3476(85)80290-0 3881580

[25] Elliot CG Morris AH Cengiz M. Pulmonary function and exercise gas exchange in survivors of adult respiratory distress syndrome. Am Rev Respir Dis (1981) 123:492–5. 7235371 10.1164/arrd.1981.123.5.492

[26] Elliott CG Rasmusson Y Crapo RO Prediction of pulmonary function abnormalities after ARDS. Am Rev Respir Dis (1987) 135:634–8. 3548507 10.1164/arrd.1987.135.3.634

[27] Ben-Abraham R Moreh O Auger-ten A Vardi A Harel R Barzilay Z Adapting prognostic respiratory variables of ARDS in children to small-scale community needs. J Crit Care (1999) 14:120–4. 10.1016/S0883-9441(99)90024-2 10527249

[28] Snyder LS Hertz MI Harmon KR Bitterman PB Failure of lung repair following acute lung injury: regulation of the fibroproliferative response (part 2). Chest (1990) 98:989–93. 10.1378/chest.98.3.733 2209162

[29] Sporn MB Robert AB Peptide growth factors in inflammation, tissue repair, and cancer. J Clin Invest (1986) 78:329–32. 10.1172/JCI112580 3525608 PMC423543

[30] Schwarz MA. Acute lung injury: cellular mechanisms and derangements. Paediatr Respir Rev (2001) 2:3–9. 10.1053/prrv.2000.0095 16263475

[31] Madtes DK Rubenfeld G Klima LD Milberg JA Steinberg KP Martin TR Elevated transforming growth factor-alpha levels in bronchoalveolar lavage fluid of patients with acute respiratory distress syndrome. Am J Respir Crit Care Med (1998) 158:424–30. 10.1164/ajrccm.158.2.9711112 9700116

[32] Armstrong L Thickett DR Mansell JP Ionescu M Hoyle E Billinghurst RC Changes in collagen turnover in early acute respiratory distress syndrome. Am J Respir Crit Care Med (1999) 160:1910–5. 10.1164/ajrccm.160.6.9811084 10588605

[33] Snyder LS Hertz MI Harmon KR Bitterman PB Failure of lung repair following acute lung injury: regulation of the fibroproliferative response (part 1). Chest (1990) 98:733–8. 10.1378/chest.98.4.989 2394149

[34] Pietra GG Rüttner JR Wüst W Glinz W The lung after trauma and shock – fine structure of the alveolar capillary barrier in 23 autopsies. J Trauma (1981) 21:454. 7230299

[35] Schnells G Voigt WH Redl H Schlag G Glatzl A. Electron-microscopic investigation of lung biopsies in patients with post-traumatic respiratory insufficiency. Acta Chir Scand (1980) 499:9. 6935901

[36] Fukuda Y Ishizaki M Masuda Y Kimura G Kawanami O Masugi Y. The role of intraalveolar fibrosis in the process of pulmonary structural remodeling in patients with diffuse alveolar damage. Am J Pathol (1987) 126:171–82. 3812636 PMC1899540

[37] Shoemaker CT Reiser KM Goetzman BW Last JA. Elevated ratios of type I/III collagen in the lungs of chronically ventilated neonates with respiratory distress. Pediatr Res (1984) 18:1176–80. 10.1203/00006450-198411000-00025 6514444

[38] Kuhn C III Boldt J King TE Jr Crouch E Vartio T McDonald JA. An immunohistochemical study of architectural remodeling and connective tissue synthesis in pulmonary fibrosis. Am Rev Respir Dis (1989) 140:1693–703. 10.1164/ajrccm/140.6.1693 2604297

[39] Ware LB Matthay MA The acute respiratory distress syndrome. N Engl J Med (2000) 342:1334–49. 10.1056/NEJM200005043421806 10793167

[40] ARDS Definition Task Force Ranieri VM Rubenfeld GD Thompson BT Ferguson ND Caldwell E Acute respiratory distress ssyndrome: the Berlin definition. JAMA (2012) 307:2526–33. 10.1016/j.medin.2012.08.010 22797452

[41] Fein A Grossman RF Jones JG Overland E Pitts L Murray JF The value of edema fluid protein measurement in patients with pulmonary edema. Am J Med (1979) 67:32–8. 10.1016/0002-9343(79)90066-4 463915

[42] Prodhan P Noviski N. Pediatric acute hypoxemic respiratory failure: management of oxygenation. J Intensive Care Med (2004) 19:140–53. 10.1177/0885066604263859 15154995

[43] The Acute Respiratory Distress Syndrome Network. Ventilation with lower tidal volumes as compared with traditional tidal volumes for acute lung injury and the respiratory distress syndrome. N Engl J Med (2000) 342:1301–8. 10.1056/NEJM200005043421801 10793162

[44] Ricard JD Dreyfuss D Saumon G. Ventilator-induced lung injury. Curr Opin Crit Care (2002) 8:12–20. 10.1097/00075198-200202000-00003 12205401

[45] Pecchiari M Monaco A Koutsoukou A D’Angelo E Plasma membrane disruptions with different modes of injurious mechanical ventilation in normal rat lungs. Crit Care Med (2012) 40:869–75. 10.1097/CCM.0b013e318232da2b 22001586

[46] Slutsky AS Lung injury caused by mechanical ventilation. Chest (1999) 116:9S–15S. 10.1378/chest.116.suppl_1.9S-a 10424561

[47] Copland IB Reynaud D Pace-Asciak C Post M. Mechanotransduction of stretch-induced prostanoid release by fetal lung epithelial cells. Am J Physiol Lung Cell Mol Physiol (2006) 291:L487–95. 10.1152/ajplung.00510.2005 16603590

[48] Torday JS Torres E Rehan VK. The role of fibroblast transdifferentiation in lung epithelial cell proliferation, differentiation, and repair in vitro. Pediatr Pathol Mol Med (2003) 22:189–207. 10.1080/15227950307732 12746170

[49] Parker JC Breen EC West JB. High vascular and airway pressures increase interstitial protein mRNA expression in isolated rat lungs. J Appl Physiol (1997) 83:1697–705. 9375341 10.1152/jappl.1997.83.5.1697

[50] Garcia CS Rocco PR Facchinetti LD Lassance RM Caruso P Deheinzelin D What increases type III procollagen mRNA levels in lung tissue: stress induced by changes in force or amplitude? Respir Physiol Neurobiol (2004) 144:59–70. 10.1016/j.resp.2004.07.023 15522703

[51] Rocco PR Dos Santos C Pelosi P. Lung parenchyma remodeling in acute respiratory distress syndrome. Minerva Anestesiol (2009) 75:730–40. 19940826

[52] Tatler AL Jenkins G TGF-beta activation and lung fibrosis. Proc Am Thorac Soc (2012) 9:130–6. 10.1513/pats.201201-003AW 22802287

[53] Tremblay LN Slutsky AS Pathogenesis of ventilator-induced lung injury: trials and tribulations. Am J Physiol Lung Cell Mol Physiol (2005) 288:L596–8. 10.1152/ajplung.00438.2004 15757952

[54] Riva DR Oliveira MB Rzezinski AF Rangel G Capelozzi VL Zin WA Recruitment maneuver in pulmonary and extrapulmonary experimental acute lung injury. Crit Care Med (2008) 36:1900–8. 10.1097/CCM.0b013e3181760e5d 18496360

[55] Steimback PW Oliveira GP Rzezinski AF Silva PL Garcia CS Rangel G Effects of frequency and inspiratory plateau pressure during recruitment meanoeuvres on lung and distal organs in acute lung injury. Intensive Care Med (2009) 35:1120–8. 10.1007/s00134-009-1439-y 19221714

[56] Zucker A Holm BA Wood LDH Crawford G Ridge K Sznajder IA Exogenous surfactant with PEEP reduces pulmonary edema and improves lung function in canine aspiration pneumonitis. J Appl Physiol (1992) 73:679–86. 1399997 10.1152/jappl.1992.73.2.679

[57] Lutz CJ Picone A Gatto LA Paskanik A Landas S Nieman G. Exogenous surfactant and positive end-expiratory pressure in the treatment of endotoxin-induced lung injury. Crit Care Med (1998) 26:1379–89. 10.1097/00003246-199808000-00025 9710098

[58] Desai LP Sinclair SE Chapman KE Hassid A Waters CM. High tidal volume mechanical ventilation with hyperoxia alters alveolar type II cell adhesion. Am J Physiol Lung Cell Mol Physiol (2007) 293:L769–78. 10.1152/ajplung.00127.2007 17601798

[59] Randolph AG. Management of acute lung injury and acute respiratory distress syndrome in children. Crit Care Med (2009) 37:2448–54. 10.1097/CCM.0b013e3181aee5dd 19531940

[60] Turi JL Cheifetz IM Acute Respiratory Failure. Resuscitation and Stabilization of the Critically ill Child. London: Springer (2009).

[61] Marraro GA. Innovative practices of ventilatory support with pediatric patients. Pediatr Crit Care Med (2003) 4:8–20. 10.1097/00130478-200301000-00003 12656536

[62] Kobayashi T Ganzuka M Taniguchi J Nitta K Murakami S. Lung lavage and surfactant replacement for hydrochloric acid aspiration in rabbits. Acta Anaesthesiol Scand (1990) 34:216–21. 10.1111/j.1399-6576.1990.tb03073.x 2111628

[63] Arikan AA Zappitelli M Goldstein SL Naipaul A Jefferson LS Loftis LL. Fluid overload is associated with impaired oxygenation and morbidity in critically ill children. Pediatr Crit Care Med (2012) 13:253. 10.1097/PCC.0b013e31822882a3 21760565

[64] Flori HR Church G Liu KD Gildengorin G Matthay MA. Positive fluid balance is associated with higher mortality and prolonged mechanical ventilation in pediatric patients with acute lung injury. Crit Care Res Pract (2011) 2011:854142. 10.1155/2011/854142 21687578 PMC3114079

[65] Foland JA Fortenberry JD Warshaw BL Pettignano R Merritt RK Heard ML Fluid overload before continuous hemofiltration and survival in critically ill children: a retrospective analysis. Crit Care Med (2004) 32:1771. 10.1097/01.CCM.0000132897.52737.49 15286557

[66] Möller JC Reiss I Schaible TF Kohl M Göpel W Fischer T Oxygenation and lung morphology in a rabbit pediatric ARDS-model under high peak pressure ventilation plus nitric oxide and surfactant compared with veno-venous ECMO. Int J Artif Organs (1999) 22:747–53. 10612302

[67] O’Rourke PP Stolar CJ Zwischenberger JB Snedecor SM Bartlett RH Extracorporeal membrane oxygenation: support for overwhelming pulmonary failure in the pediatric population. Collective experience from the extracorporeal life support organization. J Pediatr Surg (1993) 28:523–8. 10.1016/0022-3468(93)90610-W 8483064

[68] Haines NM Rycus PT Zwischenberger JB Bartlett RH Undar A. Extracorporeal Life Support Registry Report 2008: neonatal and pediatric cardiac cases. ASAIO J (2009) 55:111–6. 10.1097/MAT.0b013e318190b6f7 19092657

[69] Matalon S Holm BA Notter RH. Mitigation of pulmonary hyperoxic injury by administration of exogenous surfactant. J Appl Physiol (1987) 62:756–61. 3558235 10.1152/jappl.1987.62.2.756

[70] Loewen GM Holm BA Milanowski L Wild LM Notter RH Matalon S. Alveolar hyperoxic injury in rabbits receiving exogenous surfactant. J Appl Physiol (1989) 66:1987–92. 2496084 10.1152/jappl.1989.66.3.1087

[71] Nieman G Gatto L Paskanik A Yang B Fluck R Picone A. Surfactant replacement in the treatment of sepsis-induced adult respiratory distress syndrome in pigs. Crit Care Med (1996) 24:1025–33. 10.1097/00003246-199606000-00024 8681569

[72] Lutz C Carney D Finck C Picone A Gatto L Paskanik A Aerosolized surfactant improves pulmonary function in endotoxin-induced lung injury. Am J Respir Crit Care Med (1998) 158:840–5. 10.1164/ajrccm.158.3.9801089 9731014

[73] Eijking EP van Daal GJ Tenbrinck R Luyenduijk A Sluiters JF Hannappel E Effect of surfactant replacement on *Pneumocystis carinii* pneumonia in rats. Intensive Care Med (1990) 17:475–8. 10.1007/BF01690770 1797892

[74] Sherman MP Campbell LA Merritt TA Long WA Gunkel JH Curstedt T Effect of different surfactants on pulmonary group B streptococcal infection in premature rabbits. J Pediatr (1994) 125:939–47. 10.1016/S0022-3476(05)82013-X 7996369

[75] Tashiro K Li W-Z Yamada K Matsumoto Y Kobayashi T Surfactant replacement reverses respiratory failure induced by intratracheal endotoxin in rats. Crit Care Med (1995) 23:149–56. 10.1097/00003246-199501000-00024 8001366

[76] Karamanoukian HL Glick PL Wilcox DL Rossman JE Morin FC Holm BA Pathophysiology of congenital diaphragmatic hernia VII: inhaled nitric oxide requires exogenous surfactant therapy in the lamb model of CDH. J Pediatr Surg (1995) 30(1):1–4. 10.1016/0022-3468(95)90596-0 7722807

[77] Gommers D Hartog A van’t Veen A Lachmann B. Improved oxygenation by nitric oxide is enhanced by prior lung reaeration with surfactant, rather than positive end-expiratory pressure, in lung-lavaged rabbits. Crit Care Med (1997) 25(11):1868–73. 10.1097/00003246-199711000-00027 9366772

[78] Spragg RG Lewis JF Wurst W Hafner D Baughman RP Wewers MD Treatment of acute respiratory distress syndrome with recombinant surfactant protein C surfactant. Am J Respir Crit Care Med (2003) 167:1562–6. 10.1164/rccm.200207-782OC 12649125

[79] Günther A Schmidt R Harodt J Schmehl T Walmrath D Ruppert C Bronchoscopic administration of bovine natural surfactant in ARDS and septic shock: impact on biophysical and biochemical surfactant properties. Eur Respir J (2002) 10:797–804. 10.1183/09031936.02.00243302 12030716

[80] Walmrath D Gunther A Ghofrani HA Schermuly R Schnedier T Grimminger F Bronchoscopic surfactant administration in patients with severe adult respiratory distress syndrome and sepsis. Am J Respir Crit Care Med (1996) 154:57–62. 10.1164/ajrccm.154.1.8680699 8680699

[81] Lopez-Herce J de Lucas N Carrillo A Bustinza A Moral R. Surfactant treatment for acute respiratory distress syndrome. Arch Dis Child (1999) 80:248–52. 10.1136/adc.80.3.248 10325705 PMC1717873

[82] Hermon MM Golej J Burda G Boigner H Stoll E Vergesslich K Surfactant therapy in infants and children: three years experience in a pediatric intensive care unit. Shock (2002) 17:247–51. 10.1097/00024382-200204000-00001 11954821

[83] Herting E Moller O Schiffman JH Robertson B. Surfactant improves oxygenation in infants and children with pneumonia and acute respiratory distress syndrome. Acta Paediatr (2002) 91:1174–8. 10.1111/j.1651-2227.2002.tb00124.x 12463314

[84] Moller JC Schaible T Roll C Schiffmann JH Bindl L Schrod L Treatment with bovine surfactant in severe acute respiratory distress syndrome in children: a randomized multicenter study. Intensive Care Med (2003) 29:437–46. 12589529 10.1007/s00134-003-1650-1 PMC7095123

[85] Pappert D Busch T Gerlach H Lewandowski K Radermacher P Rossaint R Aerosolized prostacyclin versus inhaled nitric oxide in children with severe acute respiratory distress syndrome. Anesthesiology (1995) 82:1507–11. 10.1097/00000542-199506000-00020 7793662

[86] Day RW Guarin M Lynch JM Vernon DD Mean JM. Inhaled nitric oxide in children with severe lung disease: results of acute and prolonged therapy with two concentrations. Crit Care Med (1996) 24:215–21. 10.1097/00003246-199602000-00006 8605791

[87] Demirakça S Dötsch J Knothe C Magsaam J Reiter HL Bauer J Inhaled nitric oxide in neonatal and pediatric acute respiratory distress syndrome: dose response, prolonged inhalation, and weaning. Crit Care Med (1996) 24:1913–9. 10.1097/00003246-199611000-00024 8917045

[88] Okamoto K Hamaguchi M Kukita I Kikuta K Sato T. Efficacy of inhaled nitric oxide in children with ARDS. Chest (1998) 114:827–33. 10.1378/chest.114.3.827 9743174

[89] Sokol J Jacobs SE Bohn D Inhaled nitric oxide for acute hypoxic respiratory failure in children and adults: a meta-analysis. Anesth Analg (2003) 97:989–98. 10.1213/01.ANE.0000078819.48523.26 14500146

[90] Seger N Soll R. Animal derived surfactant extract for treatment of respiratory distress syndrome. Cochrane Database Syst Rev (2009) 2:CD007836. 10.1002/14651858.CD007836 19370695 PMC12296251

[91] Duffett M Choong K Ng V Randolph A Cook DJ. Surfactant therapy for acute respiratory failure in children: a systematic review and meta-analysis. Crit Care (2007) 11:R66. 10.1186/cc5944 17573963 PMC2206432

[92] Dobyns EL Cornfield DN Anas NG Fortenberry JD Tasker RC Lynch A Multicenter randomized controlled trial of the effects of inhaled nitric oxide therapy on gas exchange in children with acute hypoxemic respiratory failure. J Pediatr (1999) 134:406–12. 10.1016/S0022-3476(99)70196-4 10190913

[93] Schlag G Strohmaier W. Experimental aspiration trauma: comparison of steroid treatment versus exogenous natural surfactant. Exp Lung Res (1993) 19:397–405. 10.3109/01902149309064354 8319606

[94] McAllister F Ruan S Steele C Zheng M McKinley L Ulrich L CXCR3 and IFN protein-10 in *Pneumocystis* pneumonia. J Immunol (2006) 177(3):1846–54. 10.4049/jimmunol.177.3.1846 16849496 PMC3912555

[95] Ruan S McKinley L Zheng M Rudner X D’Souza A Kolls JK Interleukin-12 and host defense against murine *Pneumocystis* pneumonia. Infect Immun (2008) 76(5):2130–7. 10.1128/IAI.00065-08 18332204 PMC2346719

[96] Dahlem P van Aalderen WM de Neef M Dijkgraaf MG Bos AP. Randomized controlled trial of aerosolized prostacyclin therapy in children with acute lung injury. Crit Care Med (2004) 32:1055–60. 10.1097/01.CCM.0000120055.52377.BF 15071401

[97] Factor P Saldias F Ridge K Dumasius V Zabner J Jaffe HA Augmentation of lung liquid clearance via adenovirus-mediated transfer of a Na, K-ATPase beta1 subunit gene. J Clin Invest (1998) 102(7):1421–30. 10.1172/JCI3214 9769335 PMC508990

[98] Factor P Dumasius V Saldias F Brown LA Sznajder JI. Adenovirus-mediated transfer of an Na+/K+-ATPase beta1 subunit gene improves alveolar fluid clearance and survival in hyperoxic rats. Hum Gene Ther (2000) 11(16):2231–42. 10.1089/104303400750035753 11084680

[99] Ridge KM Olivera WG Saldias F Azzam Z Horowitz S Rutschman DH Alveolar type 1 cells express the alpha2 Na, K-ATPase, which contributes to lung liquid clearance. Circ Res (2003) 92(4):453–60. 10.1161/01.RES.0000059414.10360.F2 12600893

[100] Adir Y Welch LC Dumasius V Factor P Sznajder JI Ridge KM. Overexpression of the Na-K-ATPase alpha2-subunit improves lung liquid clearance during ventilation-induced lung injury. Am J Physiol Lung Cell Mol Physiol (2008) 294(6):L1233–7. 10.1152/ajplung.00076.2007 18424620

[101] Mutlu GM Sznajder JI. Mechanisms of pulmonary edema clearance. Am J Physiol Lung Cell Mol Physiol (2005) 289(5):L685–95. 10.1152/ajplung.00247.2005 16214819

[102] Otterbein LE Kolls JK Mantell LL Cook JL Alam J Choi AM. Exogenous administration of heme oxygenase-1 by gene transfer provides protection against hyperoxia-induced lung injury. J Clin Invest (1999) 103(7):1047–54. 10.1172/JCI5342 10194478 PMC408257

[103] Hashiba T Suzuki M Nagashima Y Suzuki S Inoue S Tsuburai T Adenovirus-mediated transfer of heme oxygenase-1 cDNA attenuates severe lung injury induced by the influenza virus in mice. Gene Ther (2001) 8(19):1499–507. 10.1038/sj.gt.3301540 11593363

[104] Inoue S Suzuki M Nagashima Y Suzuki S Hashiba T Tsuburai T Transfer of heme oxygenase 1 cDNA by a replication-deficient adenovirus enhances interleukin 10 production from alveolar macrophages that attenuates lipopolysaccharide-induced acute lung injury in mice. Hum Gene Ther (2001) 12(8):967–79. 10.1089/104303401750195926 11387061

[105] Buff SM Yu H McCall JN Caldwell SM Ferkol TW Flotte TR IL-10 delivery by AAV5 vector attenuates inflammation in mice with *Pseudomonas* pneumonia. Gene Ther (2010) 17(5):567–76. 10.1038/gt.2010.28 20357828

[106] Lin X Dean DA Gene therapy for ALI/ARDS. Crit Care Clin (2011) 27:705–18. 10.1016/j.ccc.2011.04.002 21742224 PMC3482940

[107] Rafat N Tonshoff B Bierhaus A Beck GC. Endothelial progenitor cells in regeneration after acute lung injury do they play a role? Am J Respir Cell Mol Biol (2013) 48:399–405. 10.1165/rcmb.2011-0132TR 23103996

[108] Verghese GM McCormick-Shannon K Mason RJ Matthay MA Hepatocyte growth factor and keratinocyte growth factor in the pulmonary edema fluid of patients with acute lung injury. Biologic and clinical significance. Am J Respir Crit Care Med (1998) 158:386–94. 10.1164/ajrccm.158.2.9711111 9700111

[109] Erjefalt JS Erjefalt I Sundler F Persson CG. In vivo restitution of airway epithelium. Cell Tissue Res (1995) 281:305–16. 10.1007/BF00583399 7648624

[110] Ulich TR Yi ES Longmuir K Yin S Biltz R Morris CF Keratinocyte growth factor is a growth factor for type II pneumocytes in vivo. J Clin Invest (1994) 93:1298–306. 10.1172/JCI117086 8132770 PMC294086

[111] Atabai K Ishigaki M Geiser T Ueki I Matthay MA Ware LB. Keratinocyte growth factor can enhance alveolar epithelial repair by nonmitogenic mechanisms. Am J Physiol Lung Cell Mol Physiol (2002) 283:L163–9. 10.1152/ajplung.00396.2001 12060573

[112] Fehrenbach H Kasper M Koslowski R Pan T Schuh D Muller M Alveolar epithelial type II cell apoptosis in vivo during resolution of keratinocyte growth factor-induced hyperplasia in the rat. Histochem Cell Biol (2000) 114:49–61. 10959822 10.1007/s004180000157

[113] Fehrenbach H Kasper M Tschernig T Pan T Schuh D Shannon JM Keratinocyte growth factor-induced hyperplasia of rat alveolar type II cells in vivo is resolved by differentiation into type I cells and by apoptosis. Eur Respir J (1999) 14:534–44. 10.1034/j.1399-3003.1999.14c10.x 10543272

[114] Gonzalez-Lopez A Albaiceta GM Repair after acute lung injury: molecular mechanisms and therapeutic opportunities. Crit Care (2012) 16:209 10.1186/cc11224 22429641 PMC3681355

[115] Bhatia M Moochhala S. Role of inflammatory mediators in the pathophysiology of acute respiratory distress syndrome. J Pathol (2004) 202:145–56. 10.1002/path.1491 14743496

[116] Kubo H. Molecular basis of lung tissue regeneration. Gen Thorac Cardiovasc Surg (2011) 59:231–44. 10.1007/s11748-010-0757-x 21484549

[117] Yee M Gelein R Mariani TJ Lawrence BP O’Reilly MA. The oxygen environment at birth specifies the population of alveolar epithelial stem cells in the adult lung. Stem Cells (2016). 10.1002/stem.2330 26891117 PMC4860080

[118] Panos RJ Rubin JS Csaky KG Aaronson SA Mason RJ Keratinocyte growth factor and hepatocyte growth factor/scatter factor are heparin-binding growth factors for alveolar type II cells in fibroblast-conditioned medium. J Clin Invest (1993) 92:969–77. 10.1172/JCI116673 7688769 PMC294937

[119] Panos RJ Bak PM Simonet WS Rubin JS Smith LJ. Intratracheal instillation of keratinocyte growth factor decreases hyperoxia-induced mortality in rats. J Clin Invest (1995) 96:2026–33. 10.1172/JCI118250 7560096 PMC185841

[120] Panos RJ Patel R Bak PM Intratracheal administration of hepatocyte growth factor/scatter factor stimulates rat alveolar type II cell proliferation in vivo. Am J Respir Cell Mol Biol (1996) 15:574–81. 10.1165/ajrcmb.15.5.8918364 8918364

[121] Crosby LM Waters CM. Epithelial repair mechanisms in the lung. Am J Physiol Lung Cell Mol Physiol (2010) 298:L715–31. 10.1152/ajplung.00361.2009 20363851 PMC2886606

[122] Chapman HA Li X Alexander JP Brumwell A Lorizio W Tan K Integrin α6β4 identifies an adult distal lung epithelial population with regenerative potential in mice. J Clin Invest (2011) 121:2855–62. 10.1172/JCI57673 21701069 PMC3223845

[123] Zheng D Limmon GV Yin L Leung NH Yu H Chow VT Regeneration of alveolar type I and II cells from Scgb1a1-expressing cells following severe pulmonary damage induced by bleomycin and influenza. PLoS One (2012) 7:e48451. 10.1371/journal.pone.0048451 23119022 PMC3485240

[124] Jain R Barkauskas CE Takeda N Bowie EJ Aghajanian H Wang Q Plasticity of Hopx(+) type I alveolar cells to regenerate type II cells in the lung. Nat Commun (2015) 6:6727. 10.1038/ncomms7727 25865356 PMC4396689

[125] Barkauskas CE Cronce MJ Rackley CR Bowie EJ Keene DR Stripp BR Type 2 alveolar cells are stem cells in adult lung. J Clin Invest (2013) 123(7):3025–36. 10.1172/JCI68782 23921127 PMC3696553

[126] Garcia O Hiatt MJ Lundin A Lee J Reddy R Navarro S Targeted type 2 alveolar cell depletion provides a dynamic functional model for lung injury repair. Am J Respir Cell Mol Biol (2016) 54(3):319–30. 10.1165/rcmb.2014-0246OC 26203800 PMC4821031

[127] Engstrom PC Holm BA Matalon S. Surfactant replacement attenuates the increase in alveolar permeability in hyperoxia. J Appl Physiol (1989) 67:688–93. 2793671 10.1152/jappl.1989.67.2.688

[128] Novotny WE Hudak BB Matalon S Holm BA Hyperoxic lung injury reduces exogenous surfactant clearance in vitro. Am J Respir Crit Care Med (1995) 151:1843–7. 10.1164/ajrccm.151.6.7767528 7767528

[129] Mulugeta S Nureki S Beers MF. Lost after translation: insights from pulmonary surfactant for understanding the role of alveolar epithelial dysfunction and cellular quality control in fibrotic lung disease. Am J Physiol Lung Cell Mol Physiol (2015) 309:L507–25. 10.1152/ajplung.00139.2015 26186947 PMC4572416

[130] Selman M Pardo A. Role of epithelial cells in idiopathic pulmonary fibrosis: from innocent targets to serial killers. Proc Am Thorac Soc (2006) 3(4):364–72. 10.1513/pats.200601-003TK 16738202

[131] Buckley S Shi W Xu W Frey MR Moats R Pardo A Increased alveolar soluble annexin V promotes lung inflammation and fibrosis. Eur Respir J (2015) 46(5):1417–29. 10.1183/09031936.00002115 26160872 PMC4767328

[132] Yanagi S Tsubouchi H Miura A Matsumoto N Nakazato M. Breakdown of epithelial barrier integrity and overdrive activation of alveolar epithelial cells in the pathogenesis of acute respiratory distress syndrome and lung fibrosis. Biomed Res Int (2015) 2015:573210. 10.1155/2015/573210 26523279 PMC4615219

[133] Cai Y Yoneda M Tomita T Kurotani R Okamoto M Kido T Transgenically-expressed secretoglobin 3A2 accelerates resolution of bleomycin-induced pulmonary fibrosis in mice. BMC Pulm Med (2015) 15:72. 10.1186/s12890-015-0065-4 26178733 PMC4504078

[134] Lindsay CD. Novel therapeutic strategies for acute lung injury induced by lung damaging agents: the potential role of growth factors as treatment options. Hum Exp Toxicol (2011) 30:701–24. 10.1177/0960327110376982 20621953

[135] Bui KCT Senadheera D Wang X Hendrickson B Friedlich P Lutzko C. Recovery of multipotent progenitors from the peripheral blood of patients requiring extracorporeal membrane oxygenation support. Am J Respir Crit Care Med (2010) 181:226–37. 10.1164/rccm.200812-1901OC 19875689

[136] Davey A McAuley DF O’Kane CM. Matrix metalloproteinases in acute lung injury: mediators of injury and drivers of repair. Eur Respir J (2011) 389:959–70. 10.1183/09031936.00032111 21565917

[137] Matthay MA Ware LB Zimmerman GA. The acute respiratory distress syndrome. J Clin Invest (2012) 122:2731–40. 10.1172/JCI60331 22850883 PMC3408735

[138] Ware LB. Pathophysiology of acute lung injury and the acute respiratory distress syndrome. Semin Respir Crit Care Med (2006) 27:337–49. 10.1055/s-2006-948288 16909368

[139] Liu KD Matthay MA. Advances in critical care for the nephrologist: acute lung injury/ARDS. Clin J Am Soc Nephrol (2008) 3(2):578–86. 10.2215/CJN.01630407 18199848 PMC6631090

[140] Budinger GR Sznajder JI. The alveolar-epithelial barrier: a target for potential therapy. Clin Chest Med (2006) 27(4):655–69. 10.1016/j.ccm.2006.06.007 17085253

[141] Smith LS Zimmerman JJ Martin TR. Mechanisms of acute respiratory distress syndrome in children and adults: a review and suggestions for future research. Pediatr Crit Care Med (2013) 14:631–43. 10.1097/PCC.0b013e318291753f 23823199

[142] Raghavendran K Pryhuber GS Chess PR Davidson BA Knight PR Notter RH. Pharmacotherapy of acute lung injury and acute respiratory distress syndrome. Curr Med Chem (2008) 15:1911–24. 10.2174/092986708785132942 18691048 PMC2636692

[143] Pryhuber GS D’Angio CT Finkelstein JN Notter RH Combined-modality therapies for lung injury. In: Notter RH Finkelstein JN Holm BA, editors. Lung injury: Mechanisms, pathophysiology, and therapy. Boca Raton: Taylor Francis Group, Inc (2005). p. 779–838.

